# Intestinal Morphogenesis in Development, Regeneration, and Disease: The Potential Utility of Intestinal Organoids for Studying Compartmentalization of the Crypt-Villus Structure

**DOI:** 10.3389/fcell.2020.593969

**Published:** 2020-10-23

**Authors:** Ohman Kwon, Tae-Su Han, Mi-Young Son

**Affiliations:** ^1^Korea Research Institute of Bioscience and Biotechnology, Daejeon, South Korea; ^2^KRIBB School of Bioscience, Korea University of Science and Technology, Daejeon, South Korea

**Keywords:** intestine, development, morphogenesis, regeneration, crypt, villus, organoid

## Abstract

The morphology and structure of the intestinal epithelium are rearranged dynamically during development, tissue regeneration, and disease progression. The most important characteristic of intestinal epithelial morphogenesis is the repetitive compartmentalized structures of crypt-villus units, which are crucial for maintaining intestinal homeostasis and functions. Abnormal structures are known to be closely associated with disease development and progression. Therefore, understanding how intestinal crypt-villus structures are formed and grown is essential for elucidating the physiological and pathophysiological roles of the intestinal epithelium. However, a critical knowledge gap in understanding the compartmentalization of the crypt-villus axis remains when using animal models, due to obvious inter-species differences and difficulty in real-time monitoring. Recently, emerging technologies such as organoid culture, lineage tracing, and single cell sequencing have enabled the assessment of the intrinsic mechanisms of intestinal epithelial morphogenesis. In this review, we discuss the latest research on the regulatory factors and signaling pathways that play a central role in the formation, maintenance, and regeneration of crypt-villus structures in the intestinal epithelium. Furthermore, we discuss how these factors and pathways play a role in development, tissue regeneration, and disease. We further explore how the current technology of three-dimensional intestinal organoids has contributed to the understanding of crypt-villus compartmentalization, highlighting new findings related to the self-organizing-process-driven initiation and propagation of crypt-villus structures. We also discuss intestinal diseases featuring abnormalities of the crypt-villus structure to provide insights for the development of novel therapeutic strategies targeting intestinal morphogenesis and crypt-villus formation.

## Introduction

The human small intestine is the primary organ responsible for the absorption and metabolism of nutrients or drugs and plays an important role in controlling diverse physiological functions such as commensal bacterium colonization and immune system regulation. The adult small intestine requires a large surface area for efficient metabolism and nutrient absorption, and the inner surface area of the adult small intestine is approximately 30 m^2^ on average (Helander and Fandriks, 2014). To form a large surface area, unique structures such as villi and microvilli are developed in the small intestine. The villi, which have a finger-shaped structure protruding toward the intestinal lumen, enormously expand the surface area of the small intestine (Walton et al., 2016). Moreover, microvilli within the brush border further amplify the surface area of the small intestine by approximately 9–16-fold (Crawley et al., 2014a; Helander and Fandriks, 2014). Appropriate development of the fingerlike protruding villus structure on the intestinal surface is crucial for nutrient and fluid absorption, allowing maintenance of homeostasis in adults and growth in children. Therefore, the reduction in inner surface area due to the defective development of villi and microvilli causes intestinal failure, including short bowel syndrome, Hirschsprung disease, and chronic intestinal pseudo-obstruction syndrome (Goulet et al., 2004).

The intestinal epithelium is rapidly self-renewing to repair damages caused by exposure to a hostile environment (Barker et al., 2007; Sato et al., 2009). To support rapid regeneration of the intestine, actively cycling intestinal stem cells (ISCs) are housed at the base of the crypt between the differentiated secretory Paneth cells (Clevers, 2013; Tan and Barker, 2014). The crypt has a flask-shaped structure located in the intervillus region and is embedded in the mesenchyme, and the actively proliferating ISCs are crypt base columnar cells that strongly express Lgr5^+^ (Barker et al., 2007). For the sustainable maintenance of the intestinal epithelium, the ISCs continuously undergo self-renewal and produce transit-amplifying cells. These cells move up the crypt-villus axis and generate every type of differentiated cell comprising the intestinal epithelium, such as enterocytes, Paneth cells, goblet cells, enteroendocrine cells, and tuft cells (Marshman et al., 2002; Clevers, 2013). After reaching the tips of the villi, cells are extruded into the lumen upon apoptosis and are replaced by new cell populations, ensuring intestinal homeostasis (Barker, 2014). Although the crypt-villus is the basic architectural unit of the intestine, there is still a lack of understanding of crypt-villus structure formation, in addition to the physiological and pathological implications of the crypt-villus structure from the perspective of functionality.

In this review, we highlight the processes responsible for the specific compartmentalization of cells into crypt-villus units in the intestinal epithelium during vertebrate intestinal development and tissue regeneration. In addition, we discuss the molecular and cellular mechanisms underlying crypt-villus morphogenesis and the pathological correlation between the compartmentalized crypt-villus structure and disease incidence.

## Villus Morphogenesis During Intestinal Development

**FIGURE 1:**
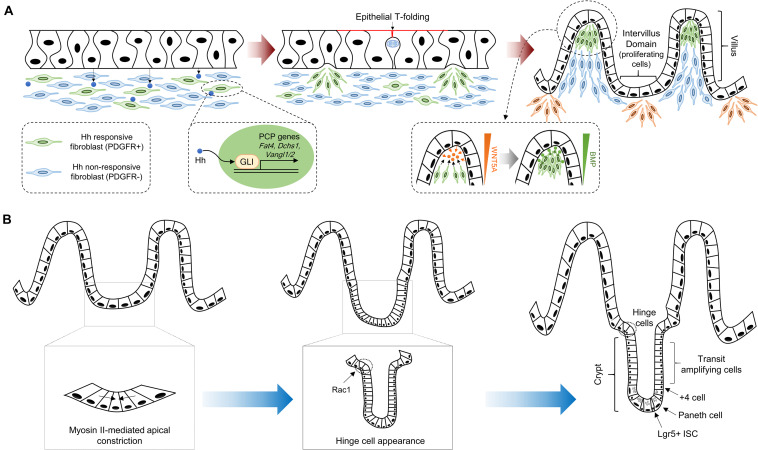
Crypt-villus morphogenesis during mouse development. **(A)** The pseudostratified intestinal epithelium secretes Hh ligands that induce expression of PCP genes, such as *Fat4*, *Dchs1*, *Vangl1*, and *Vangl2*, via GLI transcriptional factor activation. Clustered mesenchymal cells (green color) cause deformation of intestinal epithelium through apical membrane invagination with the emergence of epithelial T-folding. The clustered mesenchymal cells underneath the tip of the villus, which are formed by directional migration toward chemoattractants such as WNT5A, enforce villification and differentiation of intestinal epithelium via secretion of soluble ligands including BMPs. New mesenchymal clusters (orange color) beneath the intervillus domain lead to the second round of villification processes. **(B)** Intervillus proliferating cells transform into crypts. The initiation of crypt formation is driven by myosin II-mediated apical constriction and invagination of the crypt cells. Subsequently, hinge formation in the crypt neck is initiated to compartmentalize crypts and villus, and Rac1 controls this process through the wedge-shaped hinge cell formation. These crypts contain Lgr5 + ISCs intercalated between Paneth cells at the crypt base, and transit amplifying cells.

In vertebrates, villus morphogenesis is initiated by the synchronized development of the intestinal epithelium and mesenchyme during early embryonic development. At E14.5 in mice (weeks 8–10 in humans), the flat pseudostratified intestinal epithelium initiates morphogenesis and gives rise to villi that protrude into the intestinal lumen (Figure 1A; Spence et al., 2011a; Walton et al., 2016). To initiate villus morphogenesis, cluster formation must be carried out through mesenchymal cell condensation under the intestinal epithelium. The intestinal epithelium remains flat when mesenchymal cell clusters are formed, and villus formation is severely interrupted by the inhibition of mesenchymal cell clustering (Karlsson et al., 2000; Madison et al., 2005; Walton et al., 2012). Mesenchymal cell cluster formation is promoted by soluble ligands such as bone morphogenetic protein (Bmp), hedgehog (Hh), and platelet-derived growth factor A (Pdgf-A) secreted from the intestinal epithelium, and mesenchymal cells express receptors such as Pdgfr-α for Pdgf-A and Ptch1/2 for Hh (Karlsson et al., 2000; Madison et al., 2005; Kolterud et al., 2009; Grosse et al., 2011; Walton et al., 2012; Freddo et al., 2016). Genetic ablation of either Pdgf-A or Pdgfr-α does not inhibit the initial emergence of mesenchymal cell clusters, but subsequent mesenchymal cell cluster formation is suppressed by decreased proliferative activity of PDGFR-α-expressing mesenchymal cells (Karlsson et al., 2000). Unlike the PDGF pathway, mesenchymal cell cluster formation is severely disrupted by Hh pathway inhibition, while cluster size is increased by Hh pathway enhancement (Madison et al., 2005; Mao et al., 2010; Walton et al., 2012). This suggests that the Hh pathway is critical for the initiation of mesenchymal cell clustering and that the Pdgf pathway is necessary for cluster propagation through the activation of subsequent proliferation. Consistent with this notion, genetic deletion of the transcription factors Foxf1, Foxf2, and Foxl1, which are known to be Hh target genes, in mesenchymal cells also results in significantly reduced villus development (Kaestner et al., 1997; Ormestad et al., 2006; Madison et al., 2009).

Although the molecular mechanisms of mesenchymal cell clustering are not fully understood, increased cell adhesion and planar cell polarity (PCP) by Hh signaling are required for mesenchymal cell clustering via oriented cell movement and collective cell migration (Rao-Bhatia et al., 2020). PCP genes, including *Fat4* and *Dchs1*, were recently identified as direct target genes of Hh downstream of glioma-associated oncogene (GLI) transcription factors (Coquenlorge et al., 2019; Rao-Bhatia et al., 2020). Moreover, the activation of the *Fat4*-PCP pathway in Hh-responsive mesenchymal cells augments the formation of mesenchymal cell clustering, as this pathway controls the orientation and directional migration of mesenchymal cells toward chemoattractants such as WNT5A (Rao-Bhatia et al., 2020). The expression level of *Wnt5a* gradually increases toward the villus core, and the expression pattern of WNT5A along the villus is maintained until the late stage of development (Gregorieff et al., 2005; Rao-Bhatia et al., 2020). In addition, severe villus fusions were observed at E15.5 in both *Fat4* knockout (KO) and *Wnt5a* KO mice. Villus fusion is a requirement for *Fat4*-PCP for appropriate villus development via mesenchymal cell clustering (Cervantes et al., 2009; Rao-Bhatia et al., 2020). The mesenchymal clusters underneath the intestinal epithelial cells subsequently induce apical membrane invagination on epithelial cells, which is required for villus formation via fingerlike protrusions toward the luminal side. Intestinal epithelial cells between mesenchymal clusters form T-shaped membrane invaginations (T-folds) due to apical side constriction, which occurs via mitotic cell rounding and circumferential pressure (Nishimura et al., 2007; Kondo and Hayashi, 2013; Freddo et al., 2016). The invagination of the intestinal epithelium converts flat pseudostratified epithelium into a clearly demarcated villus structure.

After the appearance of the villus structure, proliferating cells are collected in the intervillus pockets and are subsequently confined to the crypt base (Crosnier et al., 2006; Shyer et al., 2015). The villus length is continuously increased by intervillus cell proliferation, and newly formed mesenchymal clusters between existing villi facilitate the formation of new villi. This process is repeated for several rounds, and the number of villi is approximately doubled each round (Walton et al., 2012, 2016). The architecture of the intestinal epithelium is changed by the emergence of nascent villi; therefore, the local concentration of soluble ligands for the Hh and Bmp pathways is elevated in the mesenchyme under the area of the epithelial curvature (Shyer et al., 2015). In particular, the expression levels of Bmp ligands are strongly increased in the inter-villus mesenchymal cells (Shyer et al., 2015; Walton et al., 2016). Bmp pathway activation in the intestinal epithelium suppresses Wnt signaling to promote differentiation of the progeny of proliferating cells into functional cell types, such as absorptive enterocytes, goblet cells, and enteroendocrine cells (He et al., 2004; Crosnier et al., 2006). Collectively, villification of the intestinal epithelium is regulated by the combined effects of both signaling molecules and biomechanical forces, which involve the simultaneous development of the intestinal epithelium and mesenchyme.

## Crypt Morphogenesis During Intestinal Development

In mice, crypt formation is initiated from the first week after birth (Figure 1B; Dehmer et al., 2011), but in humans, crypt formation has occurred in the intervillus regions in early gestation (weeks 11–12) (Trier and Moxey, 1979). The proliferating precryptic cells in the intervillus region are arranged in a flat sheet at P0–P2, and there is no obvious crypt-like structure (Sumigray et al., 2018). However, the apical area of crypt cells at P1 is decreased by approximately threefold compared to that at P0 (P0: 18.4 ± 0.5 mm^2^; P1: 6.2 ± 0.1 mm^2^). Additionally, the expression of myosin II-associated contractility genes, such as the myosin light chain *Myl9*, the myosin heavy chain *Myh9*, the Rho GEF *Ect2*, and the actin nucleators *Diaph2* and *Diaph3*, is increased in crypt cells compared to that in villus cells (Sumigray et al., 2018). This means that apical constriction driven by type II myosin-associated genes facilitates reduction of the apical area of crypt cells and eventually causes crypt formation by promoting epithelial sheet bending and invagination. These morphogenetic changes by apical constriction are also observed in *Xenopus* neural tube closure and *Drosophila* gastrulation (Martin et al., 2009; Rolo et al., 2009).

At P3, a broad curvature on the intestinal epithelium appears in approximately 60% of crypts, which is driven by myosin II-mediated apical constriction (Sumigray et al., 2018). The wedge-shaped crypt appearance and crypt depth continuously increase until P6 (Sumigray et al., 2018). However, at P7, an unusually shaped “hinge cell” appears at the crypt-villus boundaries and exhibits basally constricted and apically expanded morphology (Tan and Barker, 2014; Sumigray et al., 2018). Hinge cells appear to be required for maintaining the organization of the intestinal epithelium and adequate distance between villi (Sumigray et al., 2018). Interestingly, genetic ablation of Rac1 in intestinal epithelial cells impairs hinge cell formation. This is because basal constriction does not occur normally, as the attachment to the extracellular matrix (ECM) of crypt cells becomes stronger (Sumigray et al., 2018). In addition, the interaction between the ECM proteins laminin-322 and α6β4 hemidesmosomal integrin is enhanced by the increased expression of α6 and β4 integrin subunits in *Rac1* KO mice (Sumigray et al., 2018). Therefore, Rac1 plays a crucial role in regulating crypt-villus formation, in part by controlling hinge cell formation.

During the early stage of crypt development, the nascent crypts consist of just over 30 ± 9 cells (Sumigray et al., 2018). These crypts undergo continuous elongation and expansion until P14. Lgr5^+^ ISCs intercalated between Paneth cells are restricted in the crypt base, in accordance with the histological structure of mature crypts (Sato et al., 2011; Shyer et al., 2015; Yanai et al., 2017). The Lgr5^+^ ISCs undergo continuous division to maintain the morphological and functional properties of the intestinal epithelium, there by rapidly proliferating transit-amplifying (TA) cells. Furthermore, diverse types of differentiated cells are generated by asymmetric cells in a stem cell niche (Potten, 1998; Barker et al., 2007; Umar, 2010; Santos et al., 2018). Paneth cells consist of an ISC niche that excrete growth factors such as Wnt ligands and lateral activation of Notch signaling on neighboring Lgr5^+^ ISCs required for ISC maintenance (Pellegrinet et al., 2011; Sato et al., 2011). As well as, Paneth cells also contribute to the maintenance of intestinal homeostasis by sensing external stimuli including nutrient availability (Beumer and Clevers, 2016). For example, calorie restriction suppresses mTORC1 activity in Paneth cells, which induces an increase in the number of Paneth cells and Lgr5^+^ ISCs to maintain intestinal homeostasis (Yilmaz et al., 2012). It is already well-known that mTORC1 activity is important for stem niche maintenance (Kaur and Moreau, 2019), further studies are needed on the molecular mechanism of how mTORC1 activity regulates Paneth cell function needs to be elucidated. Collectively, Paneth cell consist of an essential stem niche that supports Lgr5^+^ ISCs maintenance at the bottom of the crypt where the stem cell continuously divide to produce intestinal progenitors and differentiated cells for homeostatic maintenance (Clevers, 2013; Van Der Heijden and Vermeulen, 2019).

## Crypt-Villus Morphogenesis During Tissue Regeneration

The intestinal epithelium is a physical and selectively permeable barrier that protects the host from contamination with undesirable luminal contents while simultaneously preserving the ability to absorb nutrients. Disruption of the intestinal epithelium allows the passage of microbes, microbial products, and foreign antigens into the mucosa and the host and is caused by pathogen infection, excessive uncontrolled inflammation, or vascular insults (Wang Y. et al., 2019). In addition, defects in the intestinal epithelial barrier are frequently observed in diverse pathological conditions, including inflammatory disease, ischemic events, or mechanical injuries (Quiros and Nusrat, 2019). In response to epithelial injury and to regain mucosal homeostasis, distinct repair strategies have evolved to restore the epithelial barrier and prevent translocation of undesirable luminal contents across the mucosa (Iizuka and Konno, 2011).

**FIGURE 2:**
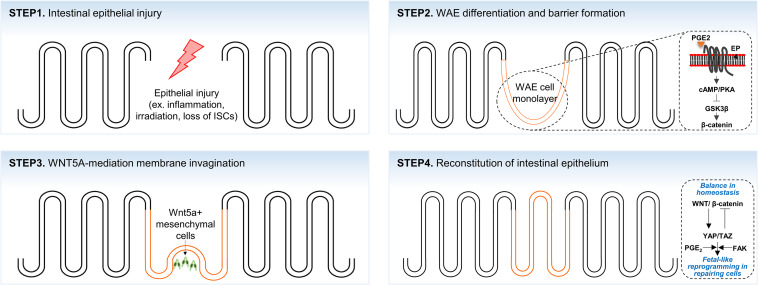
A scheme of the crypt-villus morphogenesis during tissue regeneration. Intestinal epithelium lost by intestinal injuries (STEP1) is rapidly re-epithelialized by wound-associated cell (WAE) monolayer (orange color; STEP2). Morphological transformation of the WAE monolayer into crypt-villus structure is initiated by WNT5A^+^ mesenchymal cells (STEP3) that subsequently develop to fully reconstitute the epithelium (STEP4).

In the intestinal epithelium, specialized repairing cells known as wound-associated epithelial (WAE) cells transiently emerge to establish an epithelial barrier after tissue damage (Seno et al., 2009; Miyoshi et al., 2012). Following intestinal epithelial injuries, WAE cells, which emerge from the crypts adjacent to the wounded area, rapidly migrate to cover the wound surfaces for epithelial restitution. These WAE cells are transformed into monolayer intestinal epithelium during a subsequent repair process (Figure 2; Seno et al., 2009; Miyoshi et al., 2012). Recent studies have shown that WAE differentiation and wound repair after epithelial injury are promoted by prostaglandin E2 (PGE_2_) signals through the prostaglandin E receptor 4 (EP4) on the crypts adjacent to the wound bed (Manieri et al., 2012, 2015; Miyoshi et al., 2017). In part, the generation of WAE cells by PGE_2_-EP4 signaling is due to the activation of the canonical Wnt pathway in crypt cells through nuclear β-catenin accumulation (Miyoshi et al., 2017). WAE cells are attracted by a high local concentration of PGE_2_ emanating from crypts adjacent to the wound and migrate to form an array of epithelial channel-like structures by forming a lateral, open extension toward the center of the wound area (Miyoshi et al., 2012). The wound-channel epithelium is composed of highly proliferative cells and express Axin2, a direct target of canonical Wnt signaling. This wound-channel epithelium is converted into segmented compartments similar to crypts, and epithelial morphogenesis is modulated by Wnt5a secreted from the subepithelial mesenchyme. Although the exact function of Wnt5a has not been elucidated, it is understood that Wnt5a induces multiple invagination through inhibiting the proliferation of WAE cells (Miyoshi et al., 2012). In *Wnt5a* KO mice, the wound-channel epithelium contains significantly fewer invaginations and defective crypt-like structures. This suggests that Wnt5a plays a crucial role in tissue regeneration through the proper formation and division of the wound-channel epithelium into crypts (Miyoshi et al., 2012).

The overall process of tissue regeneration through wound channel formation and epithelium invagination is similar to the epithelial response accompanied by chronic inflammation, intestinal irradiation, or ablation of Lgr5^+^ ISCs (Metcalfe et al., 2014; Nusse et al., 2018; Yui et al., 2018). Upon crypt disruption by chemical or pathogenic damage, intestinal epithelial cells around the wound area are reprogrammed into fetal-like cells. In addition, fetal signature genes are strongly enriched in repairing epithelium, whereas the expression of adult stem cell markers, such as Lgr5, Olfm4, and Lrig1, is lost (Nusse et al., 2018; Yui et al., 2018). These fetal-like repairing cells are derived from adjacent intestinal cells and migrate to the wound bed to rapidly cover the wound area. Although the molecular mechanism of injury-induced cellular reprogramming is not fully understood, it is known that the activation of the mechanosensor YAP/TAZ signaling, which is controlled by integrin-mediated bidirectional signaling between repairing cells and ECM (Lotz et al., 1997; Koivisto et al., 2014), is required for fetal-like cell reprogramming (Cai et al., 2010; Taniguchi et al., 2015; Yui et al., 2018). Consistent with this notion, transient YAP/TAZ expression has been observed to dedifferentiate committed cells back to expandable tissue-specific stem/progenitor cells (Panciera et al., 2016). Furthermore, it was recently suggested that PGE_2_ signaling upregulates the expression and transcriptional activities of YAP1 and promotes colon tissue regeneration in mice with colitis (Kim et al., 2017).

## Microvilli and Brush Border Morphogenesis

**FIGURE 3:**
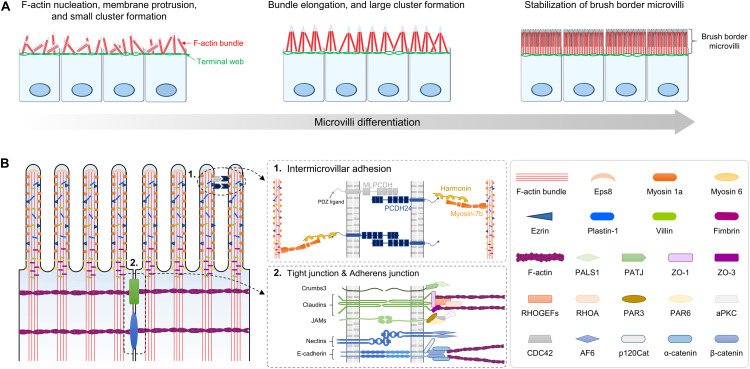
Structure and composition of microvilli during brush border development. **(A)** Small cluster of microvilli on the apical surface is formed by the formation of F-actin bundles beneath the membrane. As microvilli differentiate, clusters elongate and form large cluster, ultimately resulting in stabilized brush border. **(B)** Molecular composition and two distinctive structures of the microvilli are shown. ➀ Intermicrovillar adhesion between the distal tips of microvilli is mediated by a heterophilic complex between protocadherin-24 (PCDH24) and mucin-like protocadherin (MLPCDH). The protocadherin complex is physically linked to the actin bundle through interaction with harmonin and myosin 7b. ➁ Molecular composition of tight junctions (TJs) and adherens junction at the lateral membrane of brush border. JAMs, junctional adhesion molecules.

The brush border on the apical surface of fully differentiated enterocytes consists of highly ordered membrane protrusions, also known as microvilli. These microvilli constitute a biochemical and mechanical interface required for efficient digestion, nutrient absorption, and protection from infectious diseases (Sebe-Pedros et al., 2013; Crawley et al., 2014a; Delacour et al., 2016). While the initiation mechanism of microvilli morphogenesis remains unclear, it seems that actin filament nucleation and elongation are required to generate the force for membrane deformation (Figure 3; Pollard and Mooseker, 1981; Mooseker et al., 1982). The formation of polymerized and bundled actin microfilaments provides membrane protrusion force to drive microvillus morphogenesis. A large panel of different actin interacting proteins such as actin-capping proteins, F-actin bundling proteins, membrane-cytoskeleton crosslinking proteins, and intermicrovillar adhesion proteins is required for dense apical arrays of microvilli, known as “brush borders” (Bartles, 2000; Fehon et al., 2010; Tocchetti et al., 2010; Revenu et al., 2012; Edwards et al., 2014).

**FIGURE 4:**
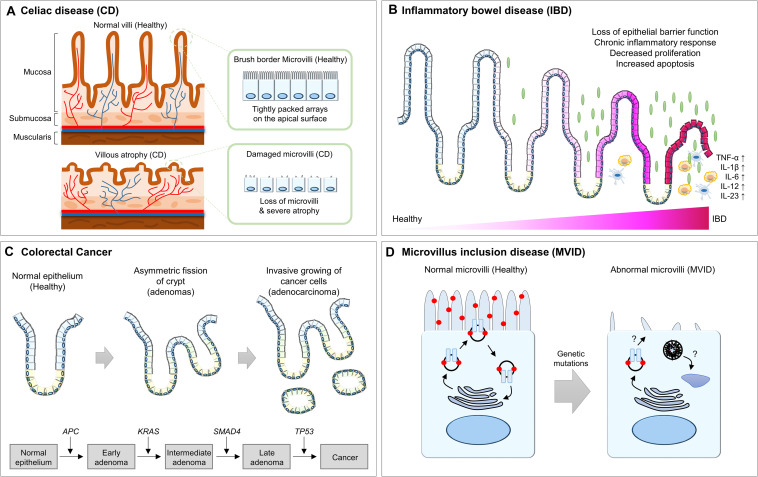
Summary of the abnormalities of intestinal epithelium in human diseases. **(A)** The healthy intestinal epithelium is compartmentalized into crypt-villus structures and consists of a tightly packed array of microvilli. In CD patients, villus atrophy causes the surface area reduction of the intestinal epithelium. **(B)** In IBD patients, chronic inflammation results in architectural abnormalities of the intestinal epithelium, which leads to distinct morphological changes within the intestinal epithelium. **(C)** In CRC patients, hyperplasia of poorly differentiated intestinal epithelial cells and invasion into the submucosal layer for metastasis is frequently observed. **(D)** In MVID patients, microvilli are distorted or absent and are instead accumulated with microvillus inclusions, which are formed by a yet unresolved mechanism. CD, celiac disease; IBD, inflammatory bowel disease; CRC, colorectal cancer; APC, adenomatous polyposis coli; MVID, microvillus inclusion disease.

The microvillar actin bundles are extended and anchored into the terminal web, which plays a critical role in the structural and mechanical stabilization of the brush border (Fath et al., 1993; Grimm-Gunter et al., 2009). In addition, the brush border microvilli have a highly ordered packing structure and uniform microvillar length (Crawley et al., 2014a, b; Delacour et al., 2016). To control brush border assembly, nascent microvilli are incorporated into existing clusters, or small clusters coalesce into larger clusters. These processes are mediated by Ca^2+^-dependent adhesion between neighboring microvilli. Ca^2+^-dependent adhesion links are formed by a heterophilic complex between protocadherin-24 (PCDH24) and mucin-like protocadherin (MLPCDH) (Crawley et al., 2014b). The protocadherins PCDH24 and MLPCDH are highly expressed and localized to the tips of microvilli through interaction with the scaffolding protein harmonin-a and the molecular motor myosin-7b. Knockdown of both protocadherins interrupts brush border assembly (Crawley et al., 2014b).

Tight junctions (TJs) are not only part of the epithelial junctional complex but are also essential components for the formation and maintenance of brush border microvilli (Saotome et al., 2004; Casaletto et al., 2011; Whiteman et al., 2014; Tilston-Lunel et al., 2016; Margolis, 2018). Thus, disruptive compositions of cell-to-cell adhesion complexes cause the appearance of short and disorganized microvilli (Saotome et al., 2004; Casaletto et al., 2011; Whiteman et al., 2014; Charrier et al., 2015). For example, *ezr* plays an essential role in assembling TJ protein complexes and linking them to the actomyocin network. *Ezr-*mutant mice exhibit disrupted apical membrane complex formation during both fetal development and adult homeostasis, and the ultrastructure of microvilli shows irregular and fused morphologies (Saotome et al., 2004; Casaletto et al., 2011). In addition, loss of the gene encoding Crumbs3 (Crb3), which is a binding protein of ezrin, also exhibits irregular fused villi and shortened microvilli as a major phenotype (Whiteman et al., 2014; Charrier et al., 2015). These results demonstrate that membrane-cytoskeleton crosslinking plays an important role in nascent microvilli stabilization and elongation.

## Crypt-Villus Morphogenesis in Intestinal Organoids

Intestinal cell lines based on two-dimensional (2D) culture technology grow flat monolayers and attach to the bottom, impeding dynamic morphogenesis such as crypt-villus formation. With the recent development of three-dimensional (3D) organoid culture methods, cell culture conditions more closely resemble *in vivo* cellular activity, and dynamic cell morphogenesis, including crypt-villus formation, is more similar to its *in vivo* counterpart (Ootani et al., 2009; Sato et al., 2009; Spence et al., 2011b; Jung et al., 2018; Wang S. et al., 2019; Zhang et al., 2020). For example, organoids contain a budding structure surrounding the central lumen; this budding domain is structurally and functionally similar to the intestinal crypt domain (Sato et al., 2009; Sato and Clevers, 2013). Similar to *in vivo* intestinal crypt domain, Lgr5^+^ ISCs, which are enriched in the budding domain, continuously divide to generate self-renewing stem cells, as well as cells terminally differentiated into enterocytes, Paneth cells, goblet cells, and enteroendocrine cells. The terminally differentiated cells migrate to the central lumen to form a villus-like domain, and eventually undergo a cell death process as they are extruded into the lumen. Furthermore, cytokines such as EGF, R-Spondin 1, and Noggin, which are essential for the formation of intestinal stem niche *in vivo*, should be included in the organoid culture medium to maintain the structural and functional properties of the intestinal organoid as the growth and differentiation of intestinal organoids is carried out by a mechanism similar to that of the intestine *in vivo* (Sato et al., 2009).

The intestinal organoids contain the crypt-villus compartment, allowing the implementation of crypt-villus morphogenesis *in vitro* (Dahl-Jensen and Grapin-Botton, 2017; Serra et al., 2019). Recently, an intestinal organoid culture technique was applied to elucidate the molecular mechanisms associated with self-organization via symmetry breaking. It has been found that these processes also play critical roles in *in vivo* intestinal morphogenesis (Serra et al., 2019). When an intestinal organoid was formed from a single ISC, it remained in a state of equilibrium, with little difference between cells until the four-cell-stage. However, in the eight- and sixteen-cell stages, the localization of YAP1 was changed in a subset of cells, and variability in the nuclear localization of YAP1 initiated symmetry breaking through a Notch-DLL1 lateral inhibition mechanism. Finally, nuclear YAP1-positive cells differentiated into Paneth cells, leading to the formation of asymmetric spheres with initial crypt-like structures. Notably, Lgr5^+^ ISCs appear by locally induced canonical Wnt signaling, and nascent crypts are reestablished by returning the stem cell niche to homeostasis (Serra et al., 2019). Organoid development from a single stem cell in a 3D culture system is considerably similar to the *in vivo* intestinal tissue regeneration process, and the molecules and signaling pathways involved in organoid development are also conserved. The initial crypt formation process occurs by a symmetry-breaking event; the crypt-villus separation process is also recapitulated in a 3D organoid culture system. When a crypt-like protrusion is generated from a round-shaped organoid, specialized wedge-shaped cells referred to as hinge cells appear at the crypt/villus boundaries (Sumigray et al., 2018; Hartl et al., 2019). These hinge cells also exist in the postnatal mouse intestine at the hinge region, and hinge formation is regulated by Rac1 activity. Upon loss of Rac1 in the mouse intestine, wedge-shaped hinge cells were absent, and the crypt/villus boundaries were ambiguous. Remarkably, genetic ablation of Rac1 in intestinal organoids reproduced abnormal compartmentalization of the crypt and villus region (Sumigray et al., 2018).

In general, 3D organoids are usually cultivated inside 3D matrices such as Matrigel. However, it is difficult to cultivate homogeneous organoids, increase the size on a macroscale, and perform experimental manipulation. In order to overcome these limitations, various types of organoid culture methods that use tissue engineering techniques have been developed (Murrow et al., 2017). For example, a technique for culture standardization of organoid culture using microcavity arrays within a polymer-hydrogel substrate (Brandenberg et al., 2020), and a culture technique to create a 3D crypt/villus structure by applying environmental stimuli (e.g., air-liquid interface) to 2D flat ISC monolayer on the transwell plate or microfluidic chip (Kim et al., 2012; Wang et al., 2015). In addition, it is also possible to generate organoids structurally and functionally similar to the *in vivo* intestinal epithelium by culturing ISCs on a scaffold that mimic crypt/villus structure using micropatterning or bioprinting technology (Wang et al., 2017; Brassard et al., 2020). These new technologies not only simulate the developmental process of crypt/villus itself, but also enable various applied research such as *in vitro* modeling of interaction among the intrinsic genetic factors and extrinsic environmental factors. Furthermore, since organoids are easily genetically manipulated though genome editing technology, genetically engineered intestinal organoids can be used to identify the roles of various genes related to crypt-villus morphogenesis (Table 1).

**TABLE 1:** Summary of gene list related to morphological abnormalities in crypt-villus structure.

Compartment	Ablated gene(s)	Intestinal phenotype	References
Villus	*Pdgf-A KO Pdgfr-*α *KO*	Abnormal GI mucosal lining misshapen villi loss of pericryptal mesenchyme	Karlsson et al., 2000
	*Shh; Ihh dKO*	Embryonic lethal reduced mesenchymal cell proliferation	Mao et al., 2010
	*Foxf1;Foxf2 dKO*	Died shortly after birth large clusters of epithelial cells and club-shaped with multilayered epithelia	Ormestad et al., 2006
	*Fat4 KO*	improperly patterned villi extensive fused villus regions failure of stromal clustering	Rao-Bhatia et al., 2020
	*Wnt5a KO*	Shortened and bifurcated intestinal lumen reduced smooth muscle layer fused villi	[Bibr B100][Bibr B14]
Crypt	*MyoIIA;MyoIIC dKO*	Disruption in apical constriction and invagination of crypt cells	Sumigray et al., 2018
	*Rac1 KO*	Flat surface and short villi crypt cell expansion deep crypts and short villi	Sumigray et al., 2018
Microvillus	*EPS8 KO*	Reduced microvillus length	Tocchetti et al., 2010
	*Espn;Vil1;Pls1 tKO*	Delay growth of microvilli sparse and misorganized microvillar actin filament bundles	Revenu et al., 2012
	*Harmonin KO*	Significant disruption in brush border morphology short and disorganized irregular microvilli	Crawley et al., 2014b
	*Ezr KO*	Short, thick, and Non-uniform architecture of microvilli disorganized and fused villi	Saotome et al., 2004
	*Crb3 KO*	Irregular and fused villi and apical membrane bleb disrupted microvilli	Whiteman et al., 2014

Intestinal organoids can be used in many ways to study crypt/villus morphogenesis, but there are still many limitations to be overcome. First, organoids currently in use consist mostly of epithelial cell. As cell-to-cell interactions with stromal cells including mesenchymal fibroblast are important in crypt/villus morphogenesis (Walton et al., 2016), it is necessary to develop organoid culture method with various cell composition. Recently, as intestinal organoid differentiation methods composed of multi-lineage cells or technologies for co-culture with various cells are actively developed, these limitations are expected to be overcome in the near future (Workman et al., 2017; Holloway et al., 2020). Second, it is necessary to develop organoid culture methods that enable changes to the environmental factors according to the crypt/villus developmental process. Currently, Matrigel is the most widely used matrix for organoid culture given its high efficiency and utility, but the batch-by-batch variation and impossible modification due to unclear chemical composition prevent its application in advanced research. Therefore, it is necessary to develop a matrix that can replace Matrigel; such new matrix should not only have a defined chemical composition and structure that can be easily modified. Currently, various matrices including hydrogel-based macromolecules have been developed (Aisenbrey and Murphy, 2020), including synthetic matrices whose physical properties change according to the crypt/villus morphogenesis of intestinal organoids (Gjorevski et al., 2016). Based on these technologies, diverse application studies have been conducted, but there are still room for improvements including their long-term stability to enable long-term culture of intestinal organoids in the future.

## Role of Gut Microbiota During Intestinal Morphogenesis

Although the basic morphology of the human intestine is formed before birth, structural and functional maturation takes place after birth, and it is known that intestinal microbiota play an important role in the structural and functional development and maturation of the human intestine (Hooper, 2004). Remarkably, germ-free mice exhibit enormous morphological defects including a decreased intestinal surface (Gordon and Bruckner-Kardoss, 1961), abnormal villus formation (Abrams et al., 1963), reduced crypt depth (Yu et al., 2016), impaired shifting of intestinal epithelial glycans (Bry et al., 1996), and poor development of the villus capillaries (Stappenbeck et al., 2002). However, these developmental defects can be compensated in adult germ-free mice upon colonization of a normal gut microbiota or a single member of gut microflora such as *B. thetaiotaomicron* (Bry et al., 1996; Hooper et al., 1999), thus suggesting that gut microbiota is essential for gut development and morphogenesis.

The bi-directional communication between intestinal epithelium and gut microbiota is important for intestinal epithelial morphogenesis, but it is still unclear why colonization of gut microbiota is necessary for intestinal epithelial morphogenesis at the molecular level. Based on recent studies, the most probable cause is that gut microbiota-derived metabolites can control the intestinal epithelial morphogenesis by affecting intestinal epithelial cell differentiation and maturation (Koh et al., 2016). Indeed, the morphological development and functional maturation of intestinal epithelium is deeply related to the diet, and in particular, the intestinal epithelium undergoes an enormous morphological changes and functional maturation during the weaning period, which is a transition from milk feeding to solid food intake (Subramanian et al., 2015). Dietary transition alters the composition and metabolism of the gut microbiota, resulting in dependence of the bi-directional interaction between gut microbiota and intestinal epithelium on dietary intake. Some of the major products from the gut microbiota are short chain fatty acids (SCFAs) including acetate, propionate, and butyrate (Cummings et al., 1987). At the cellular level, SCFAs have direct and indirect effects on the intestinal epithelial cell proliferation, differentiation, and gene expression, and butyrate reportedly suppresses colonic stem cell proliferation through receptors encoded by *Ffar3*, *Ffar2*, and *Niacr1* (Lee and Hase, 2014; Kaiko et al., 2016). Apart from SCFAs, various metabolites such as folate, bile acids, and vitamins can affect intestinal morphogenesis and functional maturation; however, further studies are required to elucidate the underlying molecular mechanisms.

## Abnormalities of the Intestinal Epithelium in Human Disease

The intestinal epithelium is compartmentalized into crypt-villus structures to maintain homeostasis and perform efficient intestinal functions. These crypt-villus units are tightly regulated by complex mechanisms, but if abnormalities occur in the crypt-villus structure, this results in various intestinal dysfunctions and disorders.

### Celiac Disease (CD)

CD, also known as celiac sprue or gluten-sensitive enteropathy, is an autoimmune disorder caused by an immune reaction triggered by ingested gluten and is by far the most common cause of villus atrophy (Aziz et al., 2017; Jansson-Knodell et al., 2018). In the small bowel, it is known that an abnormal immune response by gluten exposure causes an inflammatory response and induces villus atrophy or a significant reduction in the number of villi (Fasano, 2005; Vivas et al., 2015). Villus atrophy reduces the surface area of the intestinal epithelium and causes insufficient absorption of nutrients, micronutrients, water, and electrolytes. Therefore, multiple symptoms of intestinal dysfunctions are frequently observed in CD patients, including chronic or recurrent diarrhea, abdominal distention, anorexia, excessive nutrient deficiency, and failure to lose weight (Fasano, 2005; Vivas et al., 2015).

### Inflammatory Bowel Disease (IBD)

Although the etiology of IBD is complex, it is believed that dysregulation of the function of the intestinal epithelial barrier triggers an inappropriate immune response, leading to IBD (Kaser et al., 2010; Coskun, 2014). In fact, the loss of intestinal epithelial barrier function by altered expression of and structural changes in the intestinal TJ proteins induces epithelial damage and mucosal inflammation (Schmitz et al., 1999; Heller et al., 2005; Zeissig et al., 2007). Moreover, a disrupted epithelial architecture due to an imbalanced rate of epithelial cell proliferation and apoptosis also increases development of the chronic inflammatory response (Edelblum et al., 2006; Koch and Nusrat, 2012). IBD animal models with chronic tumor necrosis factor-[α] overexpression or dextran sulfate sodium administration exhibit significant villus atrophy and elevated epithelial cell death along the crypt-villus axis, which is most prominent at the villus tip in acute and chronic inflammation (Westbrook and Schiestl, 2010; Gunther et al., 2013; Parker et al., 2019; Lee et al., 2020). This long-lasting chronic inflammation resulting from architectural abnormalities of the intestinal epithelium leads to distinct morphological changes within the intestinal epithelium (Koch and Nusrat, 2012). This feedback loop between disrupted intestinal epithelium and chronic inflammation eventually causes the development of IBD.

### Cancer

Colorectal cancer (CRC) initially forms a benign adenoma and then develops into invasive and metastatic adenocarcinoma through the stepwise accumulation of multiple genetic and epigenetic aberrations (Fearon and Vogelstein, 1990; Grady, 2005; Nguyen and Duong, 2018). In many cases, hundreds to thousands of adenomas or adenomatous polyps are formed in CRC patients via an acquired mutation in the adenomatous polyposis coli (*APC*) gene (Fearon and Vogelstein, 1990; Kinzler and Vogelstein, 1996). These adenomas display enlarged crypts and subsequent cryptic fissions, which protrude into the intestinal lumen (Wasan et al., 1998; Taketo, 2006; Barker et al., 2007; Jones et al., 2008). For example, the representative *Apc* mutant mouse strain *Apc^Δ716^* (truncating mutation at codon 716) develops numerous polyps, not only in the small intestine, but also in the distal colon. Using this mouse model, it was determined that polyp formation is initiated by loss of heterozygosity at the *Apc* locus in proliferative zone cells, followed by the formation of an outpocket in the intestinal crypt. Interestingly, as the adenoma is covered by normal villus epithelium in the intestinal lumen, the growing adenoma does not break the crypt-villus structure. Even in relatively advanced polyps consisting of multiple villi, the normal villus epithelium is still conserved (Oshima et al., 1997; Taketo, 2006). However, malignant transformation and adenocarcinoma progresses due to continuous clonal expansion, with mutations in genes such as *KRAS*, *PIK3CA*, *SMAD4*, and *TP53*. This results in severe structural abnormalities, such as hyperplasia of poorly differentiated intestinal epithelial cells and invasion into the submucosal layer for metastasis (Humphries and Wright, 2008; Jones et al., 2008; Vogelstein et al., 2013).

### Brush Border-Related Enteropathies

The brush border on the apical surface of enterocytes consists of a tightly packed array of microvilli, which are required for supporting the apical structure and maintaining intestinal functionalities. Therefore, disrupted integrity, either through inherited or pathogen-induced defects, leads to severe disease of the intestinal epithelium. For example, microvillus inclusion disease (MVID) is a rare human congenital enteropathy that is an autosomal recessive disorder. It is characterized by chronic, severe, watery diarrhea and the insufficient absorption of necessary nutrients in newborn infants, typically beginning in the first hours to days of life. It is known that perturbed apical endosomal trafficking causes many of the abnormalities of MVID, such as defective brush border assembly, increased numbers of subapical vesicles, and the presence of microvillus inclusions in the apical region of villus enterocytes (Cutz et al., 1989; Ameen and Salas, 2000; Sherman et al., 2004; Ruemmele et al., 2006). Since apical endosomal trafficking regulates microvillus development and targeted protein delivery to the apical brush border, mutations in the *RAB8*, *RAB11*, and *MYO5B* genes associated with apical endosomal trafficking result in the MVID-like phenotype (Sato et al., 2007; Muller et al., 2008; Sobajima et al., 2014; Knowles et al., 2015). Another rare human enteropathy, congenital tufting enteropathy, is associated with mutations in the *EPCAM* and *SPINT2* genes, which result in the display of focal stacks of multiple layers of enterocytes and microvilli atrophy (Patey et al., 1997; Slae et al., 2013). In addition, a dominant mutation in *GUGY2C* and recessive mutations in *NHE3* lead to abnormalities in the apical brush border microvilli, resulting in chronic diarrhea (Janecke et al., 2015; Muller et al., 2016).

## Conclusion and Future Directions

The intestinal epithelium serves as a physical and functional barrier interfacing the external environment and luminal contents, including digestive nutrients, orally administered drugs, the microbiome, and antigens. One of the most important goals of this area of research is to understand how to establish or regenerate a normal and fully functional intestine that can successfully form crypt-villus architecture. However, there is a large gap in the knowledge surrounding morphogenesis during human intestinal development. Recent technical advances, such as the ability to culture primary intestinal tissues or organoids and the use of human PSC-derived intestinal organoids, provide new and exciting avenues for understanding the molecular mechanisms involved in human intestinal morphogenesis. To achieve a comprehensive understanding of intestinal morphogenesis and its dynamic function, multidisciplinary approaches and the development of state-of-the-art model systems offer new ways to further our understanding.

Research on intestinal epithelial morphogenesis and regeneration is rapidly expanding, but further studies related to intestinal epithelial morphogenesis and regeneration are still needed. First, through comparative analysis of normal and abnormal intestinal epithelium, key molecules and signaling pathways that are important for intestinal epithelial morphogenesis and regeneration should be identified. As high-throughput technologies such as single cell RNA sequencing and automated imaging are advancing, the understanding of the molecular mechanism of intestinal epithelial morphogenesis and regeneration is rapidly expanding. Another is the need to figure out the interactions with environmental factors such as nutrition status, mesenchymal cell composition, and gut microbiome colonization that influence intestinal epithelial morphogenesis and regeneration. Recently, bi-directional communication between intestinal epithelium and environmental factors during intestinal epithelial morphogenesis and regeneration can be recapitulated *in vitro* due to the development of the diverse platform technologies including organoid culture and co-culture in microchips. These can help the development of novel therapeutics such as probiotics that can successfully regenerate the intestinal epithelium in patients with intestinal failure or short bowel syndrome.

## Author Contributions

All authors contributed to the conception, writing, and review of the manuscript.

## Conflict of Interest

The authors declare that the research was conducted in the absence of any commercial or financial relationships that could be construed as a potential conflict of interest.
